# Veterinary Department of McGill University

**Published:** 1899-05

**Authors:** 


### VETERINARY DEPARTMENT OF McGILL UNIVERSITY.

The series of annual convocations for the conferring of degrees, which marked the close of the academic year in McGill University, opened March 30th in the Molson Hall. As upon former occasions, the Faculty of Comparative Medicine was first in the field, it being for the purpose of conferring degrees upon successful students in this Faculty that the initial meeting of convocation was held.

The meeting opened at three o'clock, the members of the teaching staff of the Faculty filing into the hall, headed by Dr. Peterson, principal of the University. The chair was taken by Mr. Hugh

McLennan, governor of the university, the governing body being also represented by Sir William C. McDonald and Mr. Samuel Finley.

After prayer the sessional report for 1898–1899 was read by the Registrar of the Faculty, Dr. Charles McEachran, in the absence of the dean, Dr. D. McEachran, who is confined to his house. The report stated that the number of students who registered was fifteen, all of whom attended the full course. Of these eight were from Canada, five from the United States, and one each from England and Japan.

The following is a list of the graduates of 1899 in their order of merit: James McGregor, J. W. Groves, G. Gellatly, Y. Kato, E. W. Hammond, J. F. Fahey.

The following is the prize list:

Veterinary medicine and surgery, won by James McGregor; cattle pathology, won by James McGregor; materia medica, won by James McGregor; anatomy, won by B. F. Humphries; physiology, won by W. C. Smith; cynology, won by J. W. Groves; chemistry, won by B. F. Humphries; botany, won by J. T. Rork.

For the best general examinations in all subjects, a silver medal, the gift of the dean, won by James McGregor.

For the best final oral examination before the Board of Examiners, won by J. W. Groves.

Extra prizes, for the best essay read before the Veterinary Medical Association—First, won by George Gellatly; second, won by James McGregor.

For the best essay read before the Society for the Study of Comparative Psychology—First, won by James McGregor; second, won by George Gellatly; third, won by E. W. Hammond.

An eloquent and evidently carefully prepared valedictory address was read by Dr. George Gellatly.

The address of the convocation to the students was delivered by Professor D. P. Penhallow. The address opened with some excellent and timely advice to those who had just completed their studies, taking thereby the all-important step in that ladder by which, he trusted, they would ultimately attain the highest measure of success in their chosen profession. The step which they had just taken marked the transition from a period of tutelage to that in which they would exercise the highest privileges of men whose minds had been trained to special services.

"There lies before you," he said, "the opportunity to take your places among those in whose hands lies the guiding force of nations; the opportunity to put to the test those powers which are the great heritage of man and which it is the peculiar function of this university to foster and call into useful service; the opportunity to assist in the great work of progressive development; and the ennobling opportunity to afford amelioration to and alleviate the sufferings of numerous helpless beings, whose peculiar position renders them specially worthy of consideration and care. Let, therefore, this all-important step be taken with due deliberation, since your future success will be determined thereby."

Professor Penhallow then pointed out the difficulties which must necessarily attend their professional career, not the least of which would be contending with the full force of a prejudice which comes down from the days by no means far distant when a scientifically trained veterinarian was unknown. He exhorted them to persevere, however, strong in the consciousness of the fact that it is the man who imparts dignity and character to his profession. He urged upon them to bear in mind ever the allegiance which they owed to their alma mater, reminding them that her approval was the surest guide.

The history of every successful university shows that in her relations to the world at large her real strength lies in her graduates. Therefore, it is only by living the lives of upright, earnest, intelligent men, having a lofty purpose in view, and consecrating to this end the best gifts of bodily and intellectual strength with which they have been endowed, and by keeping in view and respecting ever the aims and purpose of their alma mater, that true allegiance is shown.

Prof. Penhallow then referred to the Alumni Society, stating that there probably existed no more effectual method of cementing the union of the two great sections of the university body than by an annual gathering here of those who had passed hence into the sphere of active life and gathered rich stores of experience under the most diverse conditions of environment. If, at the same time, he went on, these reunions are coupled with an efficient organization, whereby the graduate body may formally deliberate upon questions of leading importance, and give an authoritative expression of their views upon all matters which virtually concern the university, they at once place themselves in the best positions for effective aid and precious advice.

Prof. Penhallow concluded by earnestly exhorting the graduates, who were now going forth upon the battle of life, to be true to their opportunities, to their university, and to themselves in their chosen work. The conclusion of Prof. Penhallow's address was marked by a hearty burst of applause.

The graduating class was then addressed by Principal Peterson, who referred to the enforced absence of Dean McEachran, who, he regretted to say, was still confined to his house. He expressed the pleasure which it gave him to look upon the excellent result of Dr. McEachran's labors in connection with the School of Comparative Medicine since its foundation, and upon his more solid work in the interest of the Dominion at large. In this connection he mentioned the valuable report in which the dean had embodied the results of a visit paid last year to corresponding centres in Great Britain and on the continent. It was, he said, asserted on the highest authority that comparative medicine offered to the young graduate as speedy a prospect of success as that offered by any other profession in the university. He added that in the United States the importance of the profession was so keenly appreciated that the Government had found it convenient to set aside large subsidies solely in its interest.

The State of New York, for example, devotes an annual subsidy of $25,000 for the support of the School of Comparative Medicine at Cornell University, while a similar subsidy was provided also for the University of Pennsylvania. Everything went to prove conclusively that the field of the veterinarian was steadily widening and extending, and he felt assured that those who were about to take up the practice of the profession would rely, in whatever centre they might elect to work, upon achieving adequate success.

The principal concluded by congratulating the gentlemen who had gained their degree, and wishing them success, prosperity, and happiness in their chosen careers.

The benediction was then pronounced and the meeting brought to a close.

